# Impact of dental caries on the quality of life of children with sickle cell anaemia in Nigeria

**DOI:** 10.4102/jphia.v16i4.1517

**Published:** 2025-12-18

**Authors:** Jacob B. Afolabi, Elizabeth O. Oziegbe, Samuel A. Adegoke, Olufemi Adefehinti, Elijah O. Oyetola, Moréniké O. Foláyan

**Affiliations:** 1 Department of Child Dental Health, Obafemi Awolowo University Teaching Hospitals’ Complex, Ile-Ife, Osun State, Nigeria; 2 Department of Child Dental Health, Faculty of Dentistry, Obafemi Awolowo University, Ile-Ife, Osun State, Nigeria; 3 Department of Paediatrics and Child Health, Faculty of Clinical Sciences, Obafemi Awolowo University, Ile-Ife, Osun State, Nigeria; 4 Department of Oral Medicine and Oral Pathology, Faculty of Dentistry, Obafemi Awolowo University, Ile-Ife, Osun State, Nigeria

**Keywords:** sickle cell anaemia, dental caries, quality of life, post treatment, children

## Abstract

**Background:**

Sickle cell anaemia, a major genetic blood disorder, is associated with serious complications, including oral health problems, which significantly impact daily living and health-related quality of life (QoL) (HRQoL).

**Aim:**

To assess the impact of untreated dental caries on the QoL of children with sickle cell anaemia (SCA).

**Setting:**

The study was carried out at the Obafemi Awolowo University Teaching Hospitals’ Complex (OAUTHC), Ile-Ife.

**Methods:**

A quasi-experimental study that included SCA children aged 8 to 16 years old with dental caries from the Paediatrics Outpatient Clinics of OAUTHC. The impact of dental caries on the oral health-related quality of life (OHRQoL) of each child was assessed pre-treatment using the Child-Oral Impact on Daily Performance (Child-OIDP) and 4 weeks post-treatment. The mean Child-OIDP scores were calculated pre- and post-treatment for the 8 different domains. A paired *t*-test was used to compare the difference in the mean Child-OIDP scores pre- and post-treatment. Statistical significance was inferred at *p* < 0.05.

**Results:**

There were 27 children with a relatively low overall pre-treatment mean Child-OIDP score of 1.63 (standard deviation [s.d. = 3.71]), which decreased to 0.37 (s.d. = 1.21) post-treatment (*p* = 0.10). The mean Child-OIDP score for the *eating* domain was significantly reduced from 0.30 (s.d. = 0.54) pre-treatment to 0.11 (s.d. = 0.42) post-treatment (*p* = 0.02).

**Conclusion:**

The overall mean Child-OIDP score was low pre-treatment, with a decline post-treatment. There was a significant reduction in the *eating* domain post-treatment.

**Contribution:**

Treatment of dental caries in children with SCA will significantly improve their oral health-related QoL (OHRQoL).

## Introduction

Sickle cell anaemia (SCA), a hereditary haemoglobinopathy prevalent in sub-Saharan Africa,^1^ significantly impacts affected children’s health-related quality of life (HRQoL), who experience lower baseline HRQoL than their healthy counterparts.^2^ This disparity extends beyond physical health, as parents of children with SCA report diminished HRQoL in domains such as self-esteem, behaviour, physical functioning and overall health perception, often more severely than the children themselves perceive.^3,4,5^ This may be linked to the challenges they face, including suboptimal physical well-being, heightened susceptibility to life-threatening complications (e.g. acute chest syndrome, recurrent infections) and prolonged treatments such as chronic transfusions.^6,7,8,9,10,11^ These factors not only impair HRQoL but also limit their ability to maintain oral hygiene, exacerbating risks for dental caries, gingivitis and periodontitis.

While poor oral health is recognised to detrimentally affect general health and quality of life (QoL),^12,13,14,15,^ limited research explores this relationship in children with SCA. Existing studies, primarily from Brazil and the United States, report no significant differences in oral health-related QoL (OHRQoL) between SCA cohorts with caries and controls, potentially because of the exclusion of participants experiencing dental pain from the cohort of study participants.^2,16^ Those with existing dental pain already have complicated caries, which may distort the research findings. In contrast, studies among healthy children demonstrate marked improvements in OHRQoL following caries treatment, suggesting that pain relief may be a critical factor.^15,17,18^ This raises questions about the unique interplay of SCA, dental pain and QoL, particularly in low-resource settings where access to care is constrained.

The Child-Oral Impact on Daily Performance (OIDP) questionnaire is a major tool widely used for the assessment of OHRQoL in children. This index considers the functional, psychological and social impacts of oral health. The tool comprises eight domains that include eating, speaking, sleeping, tooth cleaning, relaxing, emotional status, smiling, studying and social contact.^17^ These domains are important because pain is a major consequence of dental caries and other oral conditions, which negatively impact a child’s daily activities such as eating (chewing of food), sleeping and studying. Also, facial appearance plays a crucial role in communication and social interaction. The presence of dental caries, retained root or missing tooth because of caries may affect a child’s self-esteem, communication and ability to relate with peers.

Despite Nigeria’s high burden of SCA and dental diseases, no African studies have evaluated the impact of dental caries on QoL in this population or assessed post-treatment outcomes. Suburban regions, where socio-economic and healthcare disparities are pronounced, further amplify these gaps. This study, therefore, aims to investigate the influence of dental caries on the QoL of Nigerian children with SCA before and after dental intervention.

## Research methods and design

### Study design, study setting and study participants

This was a quasi-experimental study with a longitudinal, pre–post intervention aimed at evaluating how dental caries affects the QoL of children aged 8 years to 16 years living with SCA attending the Paediatrics Outpatient Clinics of the Obafemi Awolowo University Teaching Hospitals Complex, Nigeria: specifically, from the Ife Hospital Unit (IHU) and the Wesley Guild Hospital (WGH). The target age group was deliberately chosen to ensure participants could comprehend and respond accurately to the items on the study questionnaire.

Participants were recruited from a cohort of children who participated in a larger case-control study designed to identify the difference in dental caries experience, developmental defects of the enamel and teeth emergence timing of children with and without SCA. The children clinically confirmed with SCA and with visible, untreated dental caries were eligible to participate in this study. Children diagnosed with SCA had to have the presence of homozygous haemoglobin S (HbSS).

Children with other haemoglobinopathies, including compound heterozygous conditions such as HbSC (child inherits one gene for haemoglobin S and one gene for haemoglobin C) and HbE (an abnormal type of haemoglobin) or β-thalassemia, were excluded from the study to ensure genotype-specific comparisons. Additional exclusion criteria included children with a recent diagnosis of SCA, those with coexisting systemic conditions such as diabetes mellitus or hypertension, and those with intellectual disabilities that could affect their ability to participate. Moreover, children experiencing a painful crisis at the time of data collection, those who had received dental prophylaxis within the previous month, or who had undergone an emergency dental visit in the 3 months preceding the study, were also excluded to minimise potential confounding factors.

### Study procedure

Eligible study participants were required to attend the Paediatric Dentistry Outpatient Clinic of IHU or the Dentistry Outpatient Clinic at WGH. Participants were administered the Child-OIDP questionnaire independently of their caregivers, utilising visual aids such as illustrations to help explain oral conditions, particularly dental caries, to ensure comprehension. This was carried out to enable the child to document appropriately the impact of dental caries on his or her daily activities, eliminating the introduction of bias from the caregiver. The questionnaire was filled out in the waiting room. Following initial data collection, all participants received appropriate dental treatment for their carious lesions, including restorations and extractions as needed.

Four weeks after treatment, the same Child-OIDP questionnaire was administered again to the child, independent of the caregiver, to measure any change in OHRQoL. This follow-up period was selected to allow adequate healing and adaptation post-intervention, while minimising recall bias.

### Study tool

Data were collected on the age of the child at the last birthday, the sex at birth (male or female) and the socio-economic status (SES). The SES of each child was determined using a composite score derived from the educational level of one parent and the occupation of the other. In cases where only one parent was available, both the education and occupation of the living parent were used to calculate the SES.^19^ The scores were then categorised into three groups for analysis: high (comprising upper and upper-middle classes), middle (middle class) and low (lower-middle and lower classes). This classification method, widely applied in Nigeria, has been validated by Folayan et al. among parents of Nigerian children in Ile-Ife in 2003 for its reliability and accuracy.^20,21^

The Child-Oral Impact on Daily Performance (Child-OIDP) questionnaire, an instrument originally designed in English to assess OHRQoL and was initially validated for use in Thai children. Presently, it has been validated for use among children and adolescents in many countries.^14,22,23^ The tool is also used to assess the outcomes of interventions.^24,25^ It had been used in multiple studies in Africa^26^ and validated for use in Nigeria by Chukwumah et al., among children in Benin-City in 2016.^17^

The English version of the questionnaire was used to determine the impact of dental caries on the eight domains of daily performance (eating, speaking, tooth cleaning, relaxing, emotional stability, smiling, studying and engaging in social contact) by assessing the severity and frequency of the impact of dental caries in the last 3 months. Severity was rated on a four-point scale ranging from 0 (no impact) to 3 (severe impact), and frequency was similarly rated from 0 (never) to 3 (three or more times per week).^17^

The impact score for each domain was determined by multiplying the severity and frequency scores, which resulted in a value that ranged from 0 to 9 for each domain. The overall impact was determined by summing up all the scores for the eight domains; the least and maximum scores possible were 0 and 72, respectively. The score was presented in per cent- a score of 0% means no impact on QoL, while 100% indicates the highest possible impact. According to established criteria, the scores were classified into six intensity levels: none (0), very mild (1), mild (2), moderate (3–5), severe (6–8) and very severe (9).^17^

### Data analysis

The mean Child-IODP scores pre- and post-treatment were calculated for each age group, sex and SES. The difference between the Child-OIDP scores before and after treatment was determined using a paired t-test. A positive change in the score indicates an improvement in the child’s QoL. While a negative score indicated deterioration of QoL. Analyses of variance (ANOVA) were used to compare multiple means between the groups. Statistical significance was inferred at *p* < 0.05.

### Ethical considerations

Ethical clearance to conduct this study was obtained from the Obafemi Awolowo University Teaching Hospitals Complex Ethics and Research Committee (No. NHREC/27/02/2009a). Written informed consent was obtained from the parents or guardians of all prospective participants. Assent was also obtained from participants 12 years and above.

## Results

Table 1 shows the pre-treatment Child-OIDP scores among children with SCA (*N* = 27). Across all socio-demographic strata, the overall Child-OIDP scores were relatively low (range: 0.00–2.33), with substantial variability (standard deviation [s.d.] up to 4.91), though no statistically significant differences were observed. It suggests heterogeneous experiences among participants.

**TABLE 1:** Mean child-OIDP scores of caries on participants with SCA by socio-demographic variables, pre-treatment (*N* = 27).

Variables	Eating		Speaking		Cleaning		Relaxing		Emotion		Smiling		School		Contact		Overall Score	
	mean ± s.d.	*p*-value	mean ± s.d.	*p*-value	mean ± s.d.	*p*-value	mean ± s.d.	*p*-value	mean ± s.d.	*p*-value	mean ± s.d.	*p*-value	mean ± s.d.	*p*-value	mean ± s.d.	*p*-value	mean ± s.d.	*p*-value
**Age (in years)**	-	-	-	-	-	-	-	-	-	-	-	-	-	-	-	-	-	-
8–12	0.29 ± 0.47	-	0.18 ± 0.39	-	0.41 ± 0.94	-	0.24 ± 0.56	-	0.35 ±0.86	-	0.24 ± 0.56	-	0.24 ± 0.56	-	0.18 ±0.39	-	2.12 ± 4.47	-
13–16	0.00 ± 0.00	-	0.00 ± 0.00	-	0.00 ± 0.00	-	0.00 ± 0.00	-	0.00 ± 0.00	-	0.00 ± 0.00	-	0.00 ± 0.00	-	0.00 ± 0.00	-	0.00 ± 0.00	-
**Sex**	-	0.69	-	0.33	-	0.40	-	0.72	-	0.66	-	0.64	-	0.64	-	0.33	-	0.58
Male	0.22 ± 0.44	-	0.22 ± 0.44	-	0.56 ± 1.13	-	0.33 ± 0.71	-	0.33 ± 0.71	-	0.22 ± 0.44	-	0.22 ± 0.44	-	0.22 ± 0.44	-	2.33 ± 4.69	-
Female	0.33 ± 0.59	-	0.06 ± 0.24	-	0.22 ± 0.55	-	0.22 ± 0.55	-	0.17 ± 0.71	-	0.11 ± 0.47	-	0.11 ± 0.47	-	0.06 ± 0.24	-	1.28 ± 3.21	-
**Socio-economic status**	-	0.86	-	0.99	-	0.77	-	0.65	-	0.84	-	0.91	-	0.91	-	0.99	-	0.88
High	0.40 ± 0.55	-	0.20 ± 0.45	-	0.40 ± 0.89	-	0.20 ± 0.45	-	0.20 ±0.45	-	0.20 ± 0.45	-	0.20 ± 0.45	-	0.20 ±0.45	-	2.00 ± 3.94	-
Middle	0.38 ± 0.52	-	0.17 ± 0.41	-	0.50 ± 1.07	-	0.33 ± 0.82	-	0.33 ± 0.82	-	0.17 ± 0.41	-	0.17 ± 0.41	-	0.17 ± 0.41	-	2.17 ± 4.83	-
Low	0.14 ± 0.38	-	0.14 ± 0.38	-	0.29 ± 0.76	-	0.14 ± 0.38	-	0.43 ± 1.13	-	0.29 ± 0.76	-	0.29 ± 0.76	-	0.14 ± 0.38	-	1.86 ± 4.91	-

*Age-based analysis* showed that children aged 8–12 years reported measurable impacts across all domains, with the highest mean scores in cleaning (0.41 ± 0.94), emotion (0.35 ± 0.86) and eating (0.29 ± 0.47), culminating in an overall score of 2.12 ± 4.47. In contrast, participants aged 13–16 years reported no impacts (0.00 ± 0.00 across all domains).

*Sex-based comparisons* indicated marginally higher mean scores among male participants compared to female participants in most domains. For instance, male participants reported greater impacts in cleaning (0.56 ± 1.13 vs. 0.22 ± 0.55) and relaxing (0.33 ± 0.71 vs. 0.22 ± 0.55), with an overall score of 2.33 ± 4.69 for male participants versus 1.28 ± 3.21 for female participants.

*Socio-economic status* analysis revealed modest variations, with middle-SES participants reporting the highest overall impact (2.17 ± 4.83), followed by high-SES (2.00 ± 3.94) and low-SES (1.86 ± 4.91) groups. Middle-SES children scored higher in cleaning (0.50 ± 1.07) and relaxing (0.33 ± 0.82), while low-SES participants reported the lowest impacts in eating (0.14 ± 0.38).

Table 2 shows that following dental treatment, the Child-OIDP scores indicated minimal residual impacts on daily performance across all socio-demographic groups. The overall mean score post-treatment was extremely low (0.06 ± 0.24 for children aged 8–12 years and 0.00 ± 0.00 for those aged 13–16), indicating near-complete resolution of oral health-related quality of life (OHRQoL) impairments.

**TABLE 2:** Mean Child-OIDP scores of participants with sickle cell anaemia by socio-demographic variables post-treatment (*N* = 27).

Characteristics	Eating		Speaking		Cleaning		Relaxing		Emotion		Smiling		School		Contact		Overall score	
	mean ± s.d.	*p*-value	mean ± s.d.	*p*-value	mean ± s.d.	*p*-value	mean ± s.d.	*p*-value	mean ± s.d.	*p*-value	mean ± s.d.	*p*-value	mean ± s.d.	*p*-value	mean ± s.d.	*p*-value	mean ± s.d.	*p*-value
**Age (in years)**	-	-	-	-	-	-	-	-	-	-	-	-	-	-	-	-	-	-
8–12	0.06 ± 0.24	-	0.00 ± 0.00	-	0.00 ± 0.00	-	0.00 ± 0.00	-	0.00 ± 0.00	-	0.00 ± 0.00	-	0.00 ± 0.00	-	0.00 ± 0.00	-	0.06 ± 0.24	-
13–16	0.00 ± 0.00	-	0.00 ± 0.00	-	0.00 ± 0.00	-	0.00 ± 0.00	-	0.00 ± 0.00	-	0.00 ± 0.00	-	0.00 ± 0.00	-	0.00 ± 0.00	-	0.00 ± 0.00	-
**Sex**	-	0.43	-	-	-	0.49	-	0.58	-	-	-	-	-	-	-	-	-	0.37
Male	0.00 ± 0.00	-	0.00 ± 0.00	-	0.00 ± 0.00	-	0.00 ± 0.00	-	0.00 ± 0.00	-	0.00 ± 0.00	-	0.00 ± 0.00	-	0.00 ± 0.00	-	0.00 ± 0.00	-
Female	0.17 ± 0.51	-	0.00 ± 0.00	-	0.28 ± 0.96	-	0.11 ± 0.47	-	0.00 ± 0.00	-	0.00 ± 0.00	-	0.00 ± 0.00	-	0.00 ± 0.00	-	0.56 ± 1.46	-
**Socio-economic status**	-	0.63	-	-	-	-	-	-	-	-	-	-	-	-	-	-	-	0.63
High	0.20 ± 0.45	-	0.00 ± 0.00	-	0.00 ± 0.00	-	0.00 ± 0.00	-	0.00 ± 0.00	-	0.00 ± 0.00	-	0.00 ± 0.00	-	0.00 ± 0.00	-	0.20 ± 0.45	-
Middle	0.00 ± 0.00	-	0.00 ± 0.00	-	0.00 ± 0.00	-	0.00 ± 0.00	-	0.00 ± 0.00	-	0.00 ± 0.00	-	0.00 ± 0.00	-	0.00 ± 0.00	-	0.00 ± 0.00	-
Low	0.00 ± 0.00	-	0.00 ± 0.00	-	0.00 ± 0.00	-	0.00 ± 0.00	-	0.00 ± 0.00	-	0.00 ± 0.00	-	0.00 ± 0.00	-	0.00 ± 0.00	-	0.00 ± 0.00	-

*Age-based analysis* showed that children aged 8–12 years reported a negligible mean impact score of 0.06 (± 0.24), exclusively linked to the ‘eating’ domain. In contrast, adolescents (13–16 years) showed no residual impacts (0.00 ± 0.00 across all domains), suggesting age-related differences in perceived recovery.

*Sex-based comparisons* indicated that female participants exhibited slightly higher residual impacts in specific domains, such as ‘cleaning’ (0.28 ± 0.96) and ‘eating’ (0.17 ± 0.51), contributing to a marginally elevated overall score (0.56 ± 1.46). Male participants, however, reported no post-treatment impacts (0.00 ± 0.00 across all domains). Despite these trends, statistical comparisons (*p* = 0.58) confirmed no significant differences between sexes.

*Socio-economic status analysis* showed that children from high-SES backgrounds showed a minor residual impact in the ‘eating’ domain (0.20 ± 0.45), while middle- and low-SES groups reported no impacts (0.00 ± 0.00 across all domains). The lack of significant *p*-values (0.63) underscores that SES did not meaningfully influence post-treatment OHRQoL outcomes.

Table 3 shows that the mean Child-OIDP scores among 27 children with SCA revealed notable changes in the OHRQoL following dental caries treatment. Pre-treatment, the overall mean impact score was 1.63 (s.d. = 3.71), which decreased to 0.37 (s.d. = 1.21) post-treatment, though this reduction did not reach statistical significance (*p* = 0.10).

**TABLE 3:** Mean child-OIDP scores of participants with caries pre- and post-treatment by genotype (*N* = 27).

Impact domain	Sickle cell anaemia(SS)				*p*-value
	Pre-treatment (*n* = 27)		Post-treatment (*n* = 27)		
	Mean	s.d.	Mean	s.d.	
Eating	0.30	0.54	0.11	0.42	0.02
Speaking	0.11	0.32	0.00	0.00	0.08
Cleaning the mouth	0.33	0.78	0.19	0.79	0.50
Relaxing	0.26	0.59	0.07	0.39	0.06
Emotional state	0.22	0.70	0.00	0.00	0.11
Smiling	0.15	0.46	0.00	0.00	0.10
School work	0.15	0.46	0.00	0.00	0.10
Contact with persons	0.11	0.32	0.00	0.00	0.08
Overall impact score	1.63	3.71	0.37	1.21	0.10

Among specific domains, the most significant improvement was observed in *eating*, where the mean score declined from 0.30 (s.d. = 0.54) pre-treatment to 0.11 (s.d. = 0.42) post-treatment, demonstrating a statistically significant reduction (*p* = 0.02). Other domains, while showing trends toward improvement, did not achieve statistical significance. For instance, *speaking* scores decreased from 0.11 (s.d. = 0.32) to 0.00 (s.d. = 0.00; *p* = 0.08), and *relaxing* improved from 0.26 (s.d. = 0.59) to 0.07 (s.d. = 0.39; *p* = 0.06), both approaching significance. Similarly, *emotional state, smiling, schoolwork* and *social contact* domains all reported post-treatment mean scores of 0.00 (s.d. = 0.00), down from pre-treatment values of 0.22 (s.d. = 0.70), 0.15 (s.d. = 0.46), 0.15 (s.d. = 0.46) and 0.11 (s.d. = 0.32), respectively, though p-values ranged between 0.08 and 0.11. The domain *cleaning the mouth* showed minimal change, with scores shifting from 0.33 (s.d. = 0.78) to 0.19 (s.d. = 0.79; *p* = 0.50).

## Discussion

This study evaluated the impact of dental caries on the QoL of children in Nigeria with SCA before and after dental intervention. Pre-treatment, children with SCA exhibited low but variable OHRQoL impairments, particularly in domains such as eating, cleaning and emotional state. Post-treatment, overall Child-OIDP scores decreased significantly, with near-complete resolution of impacts. The most pronounced improvement occurred in the *eating* domain, that is, chewing of food was better after treatment, while other domains like *speaking, relaxing* and *emotional state* showed non-significant trends toward improvement. Notably, no socio-demographic variables significantly influenced pre-treatment or post-treatment outcomes, underscoring the universal efficacy of dental care in this cohort.

One of the strengths of the study includes its longitudinal design, which captured pre- and post-intervention changes, and the use of the validated Child-OIDP tool, ensuring culturally relevant and reliable measurements. The exclusion of confounding factors (recent dental visits, painful crises) strengthened internal validity. However, the small sample size limited statistical power, as evidenced by wide standard deviations and non-significant *p*-values for most domains. The short follow-up period (4 weeks) may also have restricted the assessment of long-term QoL outcomes. In addition, the homogeneous clinical setting (suburban Nigeria) limits generalisability to broader populations. Despite these limitations, the study highlights some important findings. Further research that recruits far more cases (SCA) and controls (normal healthy children) for comparison from the general population and a longer follow-up period is desirable.

The marked post-treatment reduction in OHRQoL impairments aligns with studies demonstrating that caries treatment improves daily functioning in healthy children.^15,17,18^ However, this contrasts with prior research in SCA cohorts from Brazil and the United States, which reported no differences in OHRQoL between SCA and non-SCA groups.^16,27^ This discrepancy may stem from methodological variations, as earlier studies excluded participants with dental pain, a key driver of QoL deficits.

In our cohort, pain relief likely explains the significant improvement in *eating*, a domain directly linked to discomfort during mastication. The significant improvement in their ability to eat following dental caries treatment illustrates how oral health is deeply intertwined with systemic disease and overall QoL. This enhancement in the eating domain reflects a complex interaction of localised oral pathology and the systemic pathophysiological context of SCA.

Dental caries, if left untreated, often advance to painful pulpitis or periapical infections. In these cases, everyday actions like chewing become painful because of the activation of sensitive A-fibre nociceptors of the trigeminal nerve fibres in decayed teeth.^28^ For children with SCA, who already endure chronic inflammation and frequent vaso-occlusive crises, the pain from dental caries is further intensified because of lower pain thresholds caused by peripheral and central sensitisation.^29^ Once the decayed teeth are restored or removed, the immediate source of nociceptive pain is eliminated, restoring the ability to chew comfortably. This relief directly translates to better eating experiences, a key determinant of OHRQoL.^30^

The benefit goes beyond the mouth. Sickle cell anaemia itself is a condition marked by heightened systemic inflammation, with elevated levels of cytokines such as IL-6 and TNF-α circulating because of recurrent ischaemic damage and haemolysis.^31^ Dental infections act as an additional inflammatory trigger, compounding the body’s inflammatory load. Treating caries helps to lower this oral source of inflammation, potentially reducing systemic inflammatory responses that worsen pain sensitivity. The combined effect of removing localised dental pain and decreasing systemic inflammation likely helps children tolerate eating better, thereby enhancing both their nutritional intake and overall QoL.

Moreover, dental pain in SCA may overwhelm already dysregulated pain modulation systems,^32^ where neurogenic inflammation and opioid receptor changes contribute to chronic pain.^33,34,35^ In this context, dental issues become more than a localised problem – they act as a compounding factor in the child’s complex pain landscape. Addressing oral pain may reset or ease this burden, reducing experiences of allodynia, where non-painful stimuli like eating become painful.

The disparity in post-treatment improvements across QoL domains among children with SCA, with marked gains in eating but limited changes in cleaning the mouth and relaxing, reflects differences in how directly these domains are influenced by dental pain, behavioural habits and systemic disease. The eating domain showed significant improvement because it is closely tied to the immediate relief of dental pain; treatment effectively removes the pain source, enabling comfortable mastication and leading to a rapid, observable improvement in eating-related QoL. In contrast, the act of cleaning the mouth involves behavioural routines and sustained effort that are not automatically restored with pain relief. Although dental discomfort may initially hinder oral hygiene, its resolution does not necessarily translate into consistent hygiene practices.

In addition, many children with SCA experience fatigue, rely heavily on caregivers and face disruptions from frequent hospitalisations. These factors, alongside lingering anxiety from past pain experiences, can impede habit formation, explaining the limited post-treatment improvement in this domain.

Relaxing, meanwhile, is shaped by the broader systemic and psychosocial burden of SCA. Despite resolving dental pain, children continue to live with chronic illness-related stressors such as anaemia, sleep disturbances, and recurrent pain episodes. These ongoing challenges affect their ability to relax, highlighting the limits of dental interventions in addressing deeper, systemic aspects of well-being. In addition, some of the limited improvements seen in non-physical domains might be attributable to sample size constraints or variability in individual experiences, which can dilute the measurable effects of dental intervention in these areas.

Furthermore, the absence of socio-demographic disparities in pre-treatment or post-treatment scores suggests that caries-related QoL impairments in SCA children are influenced more by biological factors (e.g. pain, infection) than contextual variables. This finding diverges from studies in healthy populations, where SES often correlates with oral health access and outcomes.^36^ The universal post-treatment improvement across all groups highlights the potential of targeted dental interventions to mitigate inequities in this vulnerable population.

In addition, the non-significant trends in domains like *speaking* and *emotional state* may reflect the study’s limited power rather than a true lack of effect. Larger samples could clarify whether these domains are inherently less affected by caries in SCA children or require longer observation periods to detect changes. The minimal residual impacts post-treatment further underscore the value of timely care, consistent with global calls to integrate oral health into chronic disease management.^12,13,14,15^

These findings highlight that dental interventions can substantially alleviate the oral health burden in children with SCA, particularly in pain-associated domains like eating. Clinicians managing SCA should prioritise routine dental screenings and prompt treatment to prevent QoL deterioration. Policymakers must advocate for accessible dental care in regions with high SCA prevalence, as outlined in World Health Organization (WHO) frameworks for equitable health access.^2,37^ Larger, multi-centre studies are, however, needed to validate these trends and explore subtle demographic variations. Extending follow-up periods could assess the durability of QoL improvements, while qualitative methods may capture nuanced psychosocial impacts. Collaboration between haematologists and dentists should be strengthened to optimise holistic care models for SCA patients.

## Conclusion

In conclusion, this study provides pioneering evidence that dental caries treatment significantly enhances OHRQoL in children with SCA in Nigeria, particularly in eating-related activities, irrespective of socio-demographic context. The positive shift in the eating domain following caries treatment not only underscores the tangible benefits of oral health interventions for children with SCA but also reflects the deeper, systemic impact of dental care. It emphasises the need to integrate oral health into the broader care framework for SCA. Future research could expand on these findings by investigating changes in inflammatory markers and pain processing to better understand the full impact of oral health on life with SCA.
